# An assessment of GLOBOCAN methods for deriving national estimates of cancer incidence

**DOI:** 10.2471/BLT.15.164384

**Published:** 2016-01-28

**Authors:** Sebastien Antoni, Isabelle Soerjomataram, Bjørn Møller, Freddie Bray, Jacques Ferlay

**Affiliations:** a Section of Cancer Surveillance, International Agency for Research on Cancer, 150 Cours Albert Thomas, 69372 Lyon, Cedex 08, France.; b Cancer Registry of Norway, Oslo, Norway.

## Abstract

**Objective:**

To assess the validity of the GLOBOCAN methods for deriving national estimates of cancer incidence.

**Methods:**

We obtained incidence and mortality data from Norway by region, year of diagnosis, cancer site, sex and 5-year age group for the period 1983–2012 from the NORDCAN database. Estimates for the year 2010 were derived using nine different methods from GLOBOCAN. These included the projection of national historical rates, the use of regional proxies and the combination of national mortality data with mortality to incidence ratios or relative survival proportions. We then compared the national estimates with recorded cancer incidence data.

**Findings:**

Differences between the estimates derived using different methods varied by cancer site and sex. Methods based on projections performed better where major changes in recent trends were absent. Methods based on mortality data performed less well for cancers associated with small numbers of deaths and for cancers detectable by screening. In countries with longstanding cancer registries of high quality, regional-based, or trends-based incidence estimates perform reasonably well in comparison with recorded incidence.

**Conclusion:**

Although the performance of the GLOBOCAN methods varies by cancer site and sex in this study, the results emphasize a need for more high-quality population-based cancer registries – either regional or, where practical and feasible, national registries – to describe cancer patterns and trends for planning cancer control priorities.

## Résumé

**Objectif:**

Évaluer la validité des méthodes GLOBOCAN pour établir des estimations nationales de l'incidence du cancer.

**Méthodes:**

Nous avons recueilli les données de la Norvège sur l'incidence et la mortalité par région, année de diagnostic, siège du cancer, sexe et tranche d'âge de 5 ans pour la période 1983–2012 dans la base de données NORDCAN. Les estimations pour l'année 2010 ont été établies suivant neuf méthodes GLOBOCAN différentes, dont l'extrapolation à partir de taux nationaux passés, l'utilisation d'indicateurs régionaux et la combinaison de données nationales sur la mortalité aux rapports mortalité/incidence ou aux taux de survie relatifs. Nous avons ensuite comparé les estimations nationales aux données enregistrées sur l'incidence du cancer.

**Résultats:**

Les estimations obtenues suivant différentes méthodes variaient selon le siège du cancer et le sexe. Les méthodes consistant en une extrapolation donnaient de meilleurs résultats en l'absence de changement majeur des tendances récentes. Les méthodes utilisant les données sur la mortalité s'avéraient moins fiables pour les cancers associés à un faible nombre de décès et les cancers détectables par dépistage. Dans les pays qui tenaient depuis longtemps des registres du cancer de qualité, les estimations de l'incidence d'après les indicateurs régionaux ou les tendances étaient plutôt fiables par rapport à l'incidence enregistrée.

**Conclusion:**

Bien que, dans cette étude, la fiabilité des méthodes GLOBOCAN varie selon le siège du cancer et le sexe, les résultats révèlent la nécessité de tenir davantage de registres du cancer de qualité – registres régionaux ou, lorsque cela est faisable, nationaux – afin de décrire les profils et tendances en matière de cancer et de planifier les priorités de lutte contre cette maladie.

## Resumen

**Objetivo:**

Evaluar la validez de los métodos GLOBOCAN para obtener estimaciones nacionales de la incidencia de cáncer.

**Métodos:**

Se obtuvieron datos sobre la incidencia y mortalidad en Noruega por regiones, año de diagnóstico, localización del cáncer, sexo y grupos de edades de cinco años durante el periodo comprendido entre 1983 y 2012 de la base de datos NORDCAN. Las estimaciones del año 2010 se obtuvieron utilizando nueve métodos diferentes de GLOBOCAN. Entre ellos se encontraban la proyección de tasas nacionales históricas, el uso de indicadores regionales y la combinación de datos de mortalidad nacionales con coeficientes de incidencia o porcentajes de supervivencia relativos. Posteriormente, se compararon las estimaciones nacionales con los datos de incidencia de cáncer registrados.

**Resultados:**

Las diferencias entre las estimaciones obtenidas utilizando distintos métodos variaron según la localización del cáncer y el sexo. Los métodos basados en las proyecciones mostraron mejores resultados cuando se observó una ausencia de cambios importantes en las últimas tendencias. Los métodos basados en los datos de mortalidad obtuvieron peores resultados en relación con los cánceres asociados a un menor número de fallecimientos y con los cánceres detectables en revisiones. En los países con registros de cáncer prolongados de alta calidad, las estimaciones de incidencia por regiones o por tendencias muestran resultados razonablemente buenos, en comparación con las incidencias registradas.

**Conclusión:**

A pesar de que en este estudio el rendimiento de los métodos GLOBOCAN varía según la localización del cáncer y el sexo, los resultados destacan la necesidad de obtener registros de cáncer de mejor calidad y basados en la población (ya sea a nivel regional o, cuando proceda, a nivel nacional) para describir los patrones y tendencias del cáncer para planificar las prioridades para controlarlo.

## ملخص

**الغرض:**

تقييم مدى صحة وسائل اكتشاف السرطان GLOBOCAN للخروج بتقديرات لحالات الإصابة بالسرطان على مستوى البلاد.

**الطريقة:**

لقد حصلنا على البيانات المتعلقة بحالات الإصابة والوفيات في النرويج حسب المنطقة وسنة التشخيص وموقع السرطان والجنس وفئات عمرية من 5 سنوات في الفترة من عام 1983 إلى عام 2012 من قاعدة البيانات NORDCAN. وكانت التقديرات الخاصة بعام 2010 مشتقة من استخدام تسع وسائل مختلفة من مشروع GLOBOCAN. وتضمنت هذه الوسائل تقدير المعدلات التاريخية الوطنية، والاستعانة بوكلاء إقليميين، والجمع بين بيانات الوفيات الوطنية مع نسب الوفيات جراء الإصابة أو نسب البقاء على قيد الحياة النسبية. وقمنا بعد ذلك بمقارنة التقديرات الوطنية مع بيانات حالات الإصابة المسجلة بمرض السرطان.

**النتائج:**

تباينت الاختلافات ما بين التقديرات المشتقة باستخدام وسائل مختلفة حسب موقع حدوث الإصابة بالسرطان وجنس المريض. وكان أداء الوسائل المستندة إلى التوقعات أفضل في ظل غياب أي تغييرات كبيرة في النزعات الحديثة. أما أداء الوسائل المستندة إلى بيانات الوفيات فقد كان أقل جودة لحالات الإصابة بالسرطان المرتبطة بأعداد متدنية للوفاة وبأمراض السرطان التي يمكن اكتشافها من خلال الفحص. في البلدان التي يوجد لديها سجلات لحالات الإصابة بمرض السرطان طويلة الأمد وتقوم برصد حالات الإصابة بدقة عالية على أساس إقليمي أو وفقًا للنزعات، تسير التقديرات وفقًا لأسس منطقية بالمقارنة مع حالات الإصابة المسجلة.

**الاستنتاج:**

على الرغم من أن أداء وسائل اكتشاف السرطان GLOBOCAN يتباين حسب موقع الإصابة بالسرطان وجنس المصاب في هذه الدراسة، تؤكد النتائج على الحاجة إلى إقامة سجلات للسرطان بدقة أكبر وقائمة على قطاع السكان – والتي يمكن أن تكون سجلات إقليمية أو وطنية (إذا ما توفرت إمكانية عملية وقابلة للتطبيق لإقامة تلك السجلات الوطنية) – وذلك لوصف أنماط ونزعات السرطان لكي يتم وضع التخطيط اللازم لأولويات السيطرة على السرطان.

## 摘要

**目的:**

旨在评估 GLOBOCAN 方法的有效性，从而得出国家癌症发病率估计值。

**方法:**

我们从 NORDCAN 数据库中，获得 1983–2012 年间挪威不同地区、诊断年份、癌症部位、性别以及 5 岁年龄段的发病率和死亡率数据。采用了 9 种不同的 GLOBOCAN 方法，得出 2010 年估计值。这些方法包括推断国家历史比率、使用地区代表示例以及结合国家死亡率数据和死亡率与发病率的比例或相对生存比例。我们随后将国家估计值和记录的癌症发病率数据进行比较。

**结果:**

采用因癌症部位和性别而不同的方法得出的估计值之间也有所差异。如果近期趋势中不存在主要变化，基于推断的方法则更有效。针对死亡率低的癌症或通过筛查可检测出的癌症，采用基于死亡率数据的方法效果不佳。在一些国家，相对于记录的发病率，通过长期高质量的癌症登记，基于地区或基于趋势的发病率估计值效果相当好。

**结论:**

尽管在本研究中，GLOBOCAN 方法的效果因癌症部位和性别而异，但是结果表明为描述癌症类型和趋势，以确定癌症控制工作重点，基于人群的更高质量癌症登记多多益善。这些登记可以是地区登记也可以是实用且可行的国家登记。

## Резюме

**Цель:**

Оценить пригодность методов GLOBOCAN для получения национальных прогнозов по заболеваемости раком.

**Методы:**

В Норвегии из базы данных NORDCAN были получены данные о заболеваемости и смертности, отсортированные по региону, году диагностирования, затронутому органу, полу и возрастным группам в 5-летней разбивке, относящиеся к периоду 1983–2012 гг. Прогнозы на 2010 год были получены с помощью девяти различных методов из базы данных GLOBOCAN. В их число входило проецирование национальных исторических показателей, использование приблизительных показателей по региону и объединение данных о национальной смертности со значениями отношения смертности к заболеваемости или соответствующими пропорциями выживаемости. Затем было проведено сравнение национальных прогнозов с зарегистрированными данными о заболеваемости раком.

**Результаты:**

Прогнозы, полученные с помощью разных методов, различались в зависимости от пораженного органа и пола пациента. Методы на основе проецирования позволили получить более точные данные, если в последних тенденциях отсутствовали значительные изменения. Методы, основанные на данных о смертности, позволили получить менее точные прогнозы для случаев заболевания раком, связанных с меньшим количеством летальных исходов, и для случаев заболевания, когда рак можно было выявить с помощью скринингового обследования. В странах, в которых в течение длительного времени существуют высококачественные реестры раковых заболеваний, прогнозирование заболеваемости на основании региональных данных или тенденций позволяло получить более или менее адекватные показатели по сравнению с зарегистрированной заболеваемостью.

**Вывод:**

Хотя в данном исследовании эффективность методов GLOBOCAN различалась в зависимости от пораженного органа и пола пациента, его результаты свидетельствуют о необходимости в дополнительных высококачественных реестрах раковых заболеваний среди населения на региональном или, если это целесообразно и практически осуществимо, на национальном уровне, чтобы описать модели и тенденции раковых заболеваний для определения приоритетов в борьбе с раком.

## Introduction

Cancer is among the most common causes of morbidity and mortality worldwide, with an estimated 14 million new cases and 8 million deaths in 2012, projected to rise by at least 70% by 2030.^1^ Timely and accurate cancer statistics are crucial to identify priorities for cancer control strategies at the national level. Yet, only 34 of 194 World Health Organization (WHO) Member States presently report high-quality national mortality data,^2^ while 68 countries provided high-quality incidence data for the last volume of *Cancer incidence in five continents*.^3^ As a result, many policy-makers rely on national cancer incidence and mortality estimates of variable precision to inform cancer control priorities.

GLOBOCAN, a project of the International Agency for Research on Cancer (IARC) provides estimates by cancer site and sex using the best available data in each country and several methods of estimation.^1^ Producing high-quality estimates therefore requires a dual approach of improving the reported data (developing cancer registries and civil/vital registration systems) and a continual assessment of the validity of the estimation procedures to improve the methods used.

This study focuses on the validity of the methods used in GLOBOCAN to derive national cancer incidence estimates, based on a retrospective comparison of these estimates to the observed national data in a setting with high quality cancer registry data. Although we focused on the methods most commonly used in high-income countries, we also aimed at providing insights into the validity of the methods more broadly, including methods used more predominantly in low- and middle-income countries.

## Methods

### Recorded data

To validate the nine methods used in GLOBOCAN to estimate national incidence in 2012 (GLOBOCAN 2012), long-term national and regional incidence and mortality data as well as 5-year relative survival estimates are required. Of the few countries with such data available, we selected Norway because of the consistently high quality of its cancer registry data, available nationally and by region. Cancer reporting is a legal requirement in Norway and data linkage procedures with the cause of death registry further increase the completeness of the information. For the period 2001–2005, data completeness was estimated at 98.8%, while 93.8% of the cases had been verified by examining biopsy samples under a microscope.^4^

From the Nordic cancer database NORDCAN, we extracted Norwegian incidence and mortality data by region, year of diagnosis, cancer site, sex and 5-years age group (starting at 0–4 and ending at 85+) for the period 1983–2012.^5^ We also extracted Norwegian 5-year relative survival proportions for each cancer site as well as incidence and mortality data from neighbouring countries Denmark, Finland, Iceland and Sweden.^5^ As with GLOBOCAN 2012, national population data were obtained from the United Nations^6^ while regional population data were extracted from NORDCAN.^5^

Cancer sites of the recorded cases and deaths were grouped by the codes in the *International statistical classification of diseases and related health problems, 10th revision* (ICD-10) to correspond to the sites used in GLOBOCAN. Unspecified neoplasms of the uterus (ICD-10 code C55) were reallocated to the cervix (C53) and corpus uteri (C54) according to the respective proportions of these two sites in the different datasets.^7^

We computed the number of cases by sex and cancer site in Norway in 2010 as the average of the recorded cancer cases between 2009 and 2011 to define a gold standard for comparisons. We then applied each of the nine methods used in GLOBOCAN 2012 to estimate the number of cancer cases in Norway in 2010, by sex and cancer site, and compared these estimates with the gold standard.

### Estimation methods

**Fig. 1:**
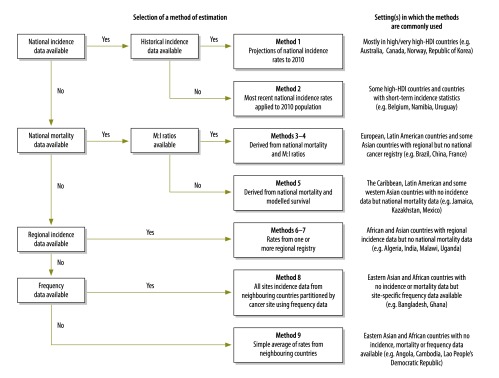
Method selection algorithm and the setting(s) in which methods were most commonly applied when estimating cancer incidence in GLOBOCAN 2012

**Fig. 2:**
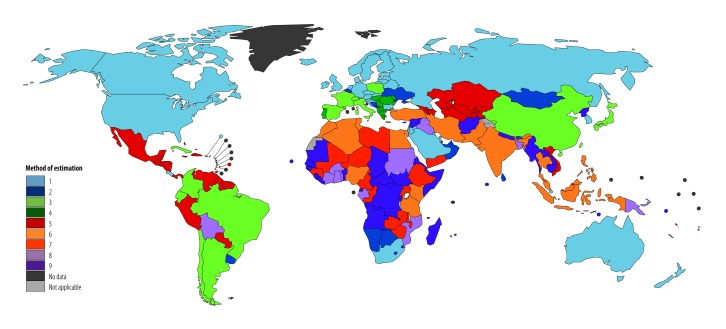
Methods of national cancer incidence estimation used for 184 countries for the GLOBOCAN 2012 project

The GLOBOCAN methods are summarized in Fig. 1, together with the algorithm used to select them in GLOBOCAN based on the availability of data in each country. More details can be found elsewhere.^1^^,^^8^ Fig. 2 illustrates which method was used for each country within the GLOBOCAN 2012 project.

HDI: Human Development Index; M:I mortality:incidence.

Notes: For Method 1, two projections were done. Method 1A used NORDPRED (5-year intervals, > 15 years of data) and method 1B used DEPPRED (annual, < 10 years of data).

Method 2 also used for cancer sites with stabilizing rates following large temporal variations (for example due to screening).

Method 3 used M:I ratios from regional registries and method 4 used data from neighbouring countries.

Method 6 used rates from one registry and method 7 used weighted rates from multiple registries.

The data required for each of the nine methods are summarized in Table 1.The methods used may produce under- or overestimates at different cancer sites. Therefore, presenting an overall number of cases based on the sum of site-specific numbers could be misleading, if aggregated overestimates and underestimates cancel each other out. We thus report separately the total number of cases underestimated and overestimated for each method. These were then aggregated to assess the differences between the results and the Norwegian recorded data.

**Table 1:** Required conditions for reliable estimations for each of the nine methods used to estimate cancer incidence in GLOBOCAN 2012

Method	Data required to use the method	Conditions required for reliable estimations
1	Historical national incidence data	– Availability of robust data on cases/population size
		– Recent incidence trends continue into near future
2	Recent national incidence data	– Availability of robust data on cases/population size
		– Stable incidence rates in near future
3	National mortality data and M:I ratios from regional registries within the country	– Availability of robust data on cases/deaths
		– Trends in incidence, mortality and survival are relatively stable over time
		– Case fatality in combined regions representative nationally
4	National mortality data and M:I ratios from registries in neighbouring countries	– Availability of robust data on cases/deaths
		– Trends in incidence, mortality and survival are relatively stable over time
		– Case fatality in combined neighbouring countries representative nationally
5	National mortality and 5-year relative survival data	– Availability of robust data on deaths and survival
		– Trends in incidence, mortality and survival are relatively stable over time
		– Five-year survival proportion a reasonable proxy for clinical cure
6	Rates from one regional registry within the country	– Availability of robust data on cases/population size
		– Incidence rates in single region representative nationally
7	Rates from multiple regional registries within the country	– Availability of robust data on cases/population size
		– Incidence rates in combined regions representative nationally
8	Data from all sites by age and sex and frequency data by cancer site	– Availability of robust data on total cancer cases
		– Total cases and cancer-specific frequencies representative nationally
9	Data from neighbouring countries	– Availability of robust data on cases/population size
		– Incidence rates in combined neighbouring countries representative nationally

M:I: mortality:incidence.

All analyses were performed using the R software package (The R Project for Statistical Computing, Vienna, Austria).

#### Method 1

Method 1 is based on projections of incidence rates. We performed two projections: (i) for 1A we used the computer program NORDPRED^9^ and applied long-term data (1983–2007) and; (ii) for 1B we used the computer program DEPPRED^10^ and applied medium-term data (1998–2007).

#### Methods 2 to 7

For methods 2 to 7, we used incidence and/or mortality data from 2003–2007 to simulate a real-life situation where data from the latest volume of *Cancer incidence in five continents (Vol. X)* would be used.^3^ The 2010 Norwegian mortality data used in methods 3 to 5 were estimated as in GLOBOCAN 2012 by projecting rates for the period 1988–2007 to 2008–2012.

In method 3, mortality:incidence (M:I) ratios from regional registries are used as a proxy for national case-fatality rates. National incidence rates can then be inferred from national mortality data along with the M:I ratio. This is useful where regional registries are numerous but not necessarily nationally representative, as in Italy^11^ or Japan.^12^ Where no such regional population-based data are available, data from neighbouring countries can be used (method 4). To generate the M:I ratios used in method 3, we included recorded cancer cases and deaths from all regions of Norway except for the south-eastern region (that includes Oslo). In some high-income countries (e.g. France or Japan) national estimates are derived from regional cancer registry data that do not cover the capital city which is usually highly populated. We also included recorded cases and deaths from other Nordic countries for cancer sites with less than a hundred deaths in Norway (e.g. cancers of the larynx, testis and thyroid and Hodgkin lymphoma).

Method 5 estimates national cancer incidence by using national mortality and 5-year relative cancer survival data, using the equation:

*M* = *I*(1–*S*) (1)

where *M* is the mortality rate, *I* is incidence rate and *S* is the 5-year relative survival proportion.

Method 6 was based on incidence data from the northern and western regions of Norway, while we selected the south-eastern region (including Oslo) for method 7. For GLOBOCAN estimations, regional incidence data are often only available from large cities, particularly in low- and middle-income countries (e.g. Uganda, Zimbabwe).

#### Methods 8 and 9

The incidence rates from neighbouring countries used in methods 8 and 9 were computed using data from Nordic countries for the period 2009–2011.

## Results

**Fig. 3:**
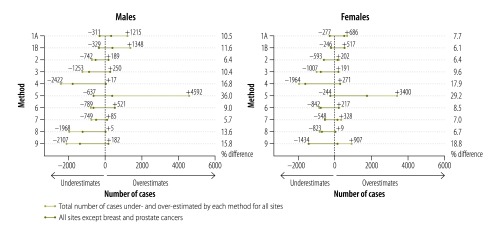
Observed and estimated cancer incidence in Norway, 2010

Note: Observed data obtained from the Norwegian Cancer Registry in 2010. Estimates are based on the nine methods used in GLOBOCAN 2012.

In 2010, 14 507 new cancer cases were recorded in Norwegian men and 12 466 in women. Our corresponding estimates, based on GLOBOCAN methods, differed by 5.7–18.8% (834 to 2341 cases) from the observed data (excluding method 5). Fig. 3 summarizes the sex-specific numerical differences according to each method, with under- and overestimates reported separately, as well as the overall difference as a percentage with observed data.

Comparing incidence estimates to observed data across cancer sites by sex, estimates based on data from one regional cancer registry (method 7) performed best in men (mean of 5.7%, or 834 difference between estimated and observed cases), while projection of medium-term historical rates (method 1B) performed best in women (mean: 6.1% difference; 763 cases). When considering both sexes together, and among the methods usually used in high-income countries (methods 1 to 4), the most recent recorded rates applied to 2010 population (method 2) performed well with a 6.4% (1726 cases) difference between observed and estimated cases. However, when prostate and breast cancers were excluded, projection of rates (methods 1A and 1B) produced very similar overall estimates to those from method 2 (at most a 5.0% (723 cases, 1B) and 7.7% (958 cases, 1A) difference; Fig. 3). Apart from methods 1A, 1B and 5, all methods tended to underestimate the total number of cases.

**Fig. 4:**
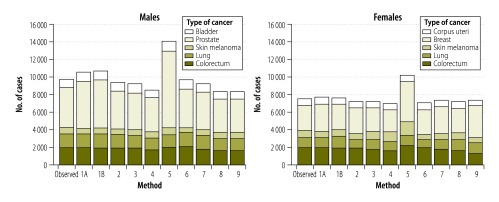
Observed and estimated cancer incidence for the five most common cancers in Norway, 2010

Notes: Observed data obtained from the Norwegian Cancer Registry in 2010. Estimates are based on the nine methods used in GLOBOCAN 2012.

**Fig. 5:**
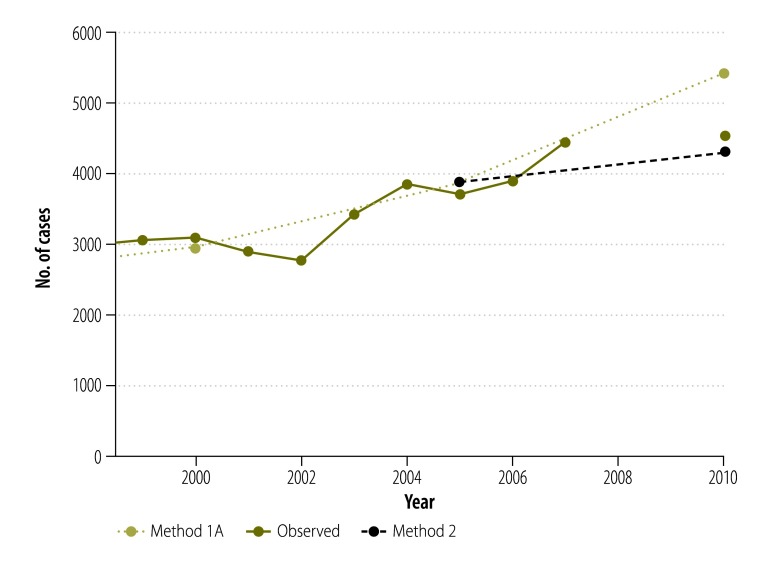
Observed and estimated incidence of prostate cancer (C61), Norway, 1999–2010

Notes: Observed data obtained from the Norwegian Cancer Registry in 2010. Estimates are based on methods 1A and 2 used in GLOBOCAN 2012.

**Fig. 6:**
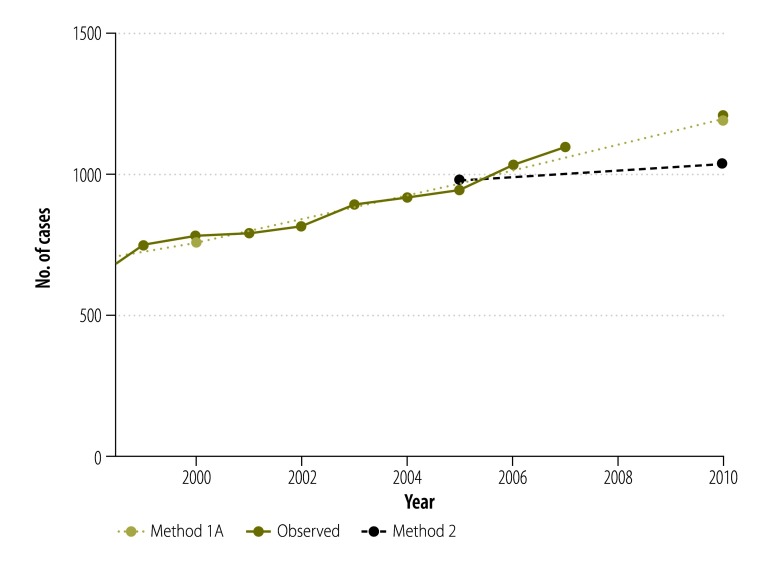
Observed and estimated incidence of lung cancer (C33–34) in females, Norway, 1999–2010

Notes: Observed data obtained from the Norwegian Cancer Registry in 2010. Estimates are based on methods 1A and 2 used in GLOBOCAN 2012.

Our estimates by cancer sites show variability in the performance of the different methods (Fig. 4). Overall, methods commonly used in high-income countries performed quite well in estimating recent cancer incidence in Norway. Method 1A produced the closest estimates to observed data for lung cancer in both men and women (−0.5%; −7 cases and −1.1%; −13 cases, respectively). It also performed well for colorectal cancer in men (−0.1%; −1 case) and women (+2.4%; +45 cases). On the other hand, prostate cancer cases were overestimated by this method (+19.4%; +881 cases). Method 2 performed better than method 1A for breast (+2.3%; +67 cases) and prostate (−5.1%; −231 cases; Fig. 5) cancers. Method 2 estimates for lung cancer were satisfactory in men (+1.2%; +18 cases) but less so in women (−13.9%; −168 cases; Fig. 6). Methods 3 and 4 generally produced underestimations at major cancer sites except for melanoma of skin in women (+17.4%; +139 cases and +34.0%; +271 cases using methods 3 and 4, respectively). These two methods performed less well for rare cancers (e.g. gallbladder cancer or Hodgkin lymphoma) or those with a good prognosis (e.g. testis or thyroid cancers; Table 2 and Table 3).

**Table 2:** Cancer incidence in males (Norwegian Cancer Registry, 2010) and estimates using GLOBOCAN methods

Site (ICD-10 code)	No. of observed cases	No. of estimated cases (% difference from observed cases)									
		Method 1A	Method 1B	Method 2	Method 3	Method 4	Method 5	Method 6	Method 7	Method 8	Method 9
Head and neck (C00–14)	324	279 (−13.9)	288 (−11.1)	287 (−11.4)	306 (−5.6)	246 (−24.1)	221 (−31.8)	292 (−9.9)	298 (−8.0)	291 (−10.2)	335 (3.4)
Oesophagus (C15)	168	159 (−5.4)	160 (−4.8)	154 (−8.3)	181 (7.7)	170 (1.2)	182 (8.3)	146 (−13.1)	166 (−1.2)	155 (−7.7)	217 (29.2)
Stomach (C16)	293	296 (1.0)	294 (0.3)	355 (21.2)	281 (−4.1)	269 (−8.2)	276 (−5.8)	431 (47.1)	296 (1.0)	238 (−18.8)	297 (1.4)
Colon-rectum (C18–21)	1948	1947 (0.0)	1882 (−3.4)	1895 (−2.7)	1834 (−5.8)	1668 (−14.4)	1994 (2.4)	2083 (6.9)	1797 (−7.8)	1617 (−17.0)	1428 (−26.7)
Liver (C22)	120	105 (−12.5)	98 (−18.3)	94 (−21.7)	100 (−16.7)	99 (−17.5)	112 (−6.7)	91 (−24.2)	94 (−21.7)	113 (−5.8)	193 (60.8)
Gallbladder (C23–24)	73	76 (4.1)	64 (−12.3)	67 (−8.2)	43 (−41.1)	25 (−65.8)	28 (−61.6)	68 (−6.8)	71 (−2.7)	60 (−17.8)	89 (21.9)
Pancreas (C25)	332	377 (13.6)	385 (16.0)	352 (6.0)	376 (13.3)	309 (−6.9)	367 (10.5)	343 (3.3)	355 (6.9)	281 (−15.4)	329 (−0.9)
Larynx (C32)	97	89 (−8.3)	82 (−15.5)	107 (10.3)	113 (16.5)^a^	112 (15.5)	119 (22.7)	110 (13.4)	113 (16.5)	83 (−14.4)	104 (7.2)
Lung (C33–34)	1561	1554 (−0.5)	1617 (3.6)	1579 (1.2)	1494 (−4.3)	1338 (−14.3)	1439 (−7.8)	1654 (6.0)	1549 (−0.8)	1357 (−13.1)	1357 (−13.1)
Melanoma of skin (C43)	773	616 (−20.3)	672 (−13.1)	592 (−23.4)	669 (−13.5)	726 (−6.1)	815 (5.4)	503 (−34.9)	646 (−16.4)	741 (−4.1)	578 (−25.2)
Prostate (C61)	4533	5414 (19.4)	5487 (21.1)	4302 (−5.1)	4165 (−8.1)	3897 (−14.0)	8741 (92.8)	4397 (−3.0)	4286 (−5.5)	3805 (−16.1)	3815 (−15.8)
Testis (C62)	288	299 (3.8)	303 (5.2)	272 (−5.6)	229 (−20.5)^a^	230 (−20.1)	172 (−40.3)	282 (−2.1)	256 (−11.1)	232 (−19.4)	185 (−35.8)
Kidney (C64–66)	484	463 (−4.3)	452 (−6.6)	407 (−15.9)	404 (−16.5)	296 (−38.8)	418 (−13.6)	405 (−16.3)	406 (−16.1)	428 (−11.6)	400 (−17.4)
Bladder (C67)	938	1045 (11.4)	1050 (11.9)	1000 (6.6)	1071 (14.2)	843 (−10.1)	1078 (14.9)	1060 (13.0)	956 (1.9)	809 (−13.8)	924 (−1.5)
Brain (C70–72)	518	628 (21.2)	625 (20.7)	530 (2.3)	562 (8.5)	368 (−29.0)	563 (8.7)	527 (1.7)	515 (−0.6)	452 (−12.7)	412 (−20.5)
Thyroid (C73)	77	80 (3.9)	87 (13.0)	67 (−13.0)	38 (−50.7)^a^	38 (−50.7)	70 (−9.1)	61 (−20.8)	71 (−7.8)	65 (−15.6)	87 (13.0)
Hodgkin lymphoma (C81)	73	94 (28.8)	73 (0.0)	76 (4.1)	54 (−26.0)^a^	47 (−35.6)	45 (−38.4)	63 (−13.7)	80 (9.6)	62 (−15.1)	63 (−13.7)
Non-Hodgkin lymphoma (C82–85, C96)	499	483 (−3.2)	485 (−2.8)	451 (−9.6)	426 (−14.6)	391 (−21.6)	444 (−11.0)	420 (−15.8)	473 (−5.2)	441 (−11.6)	455 (−8.8)
Multiple myeloma (C88, C90)	218	210 (−3.7)	211 (−3.2)	202 (−7.3)	186 (−14.7)	169 (−22.5)	216 (−0.9)	187 (−14.2)	209 (−4.1)	194 (−11.0)	162 (−25.7)
Leukaemia (C91–95)	363	339 (−6.6)	344 (−5.2)	336 (−7.4)	310 (−14.6)	292 (−19.6)	403 (11.0)	290 (−20.1)	361 (−0.5)	368 (1.4)	313 (−13.8)
Other and unspecified	827	858 (3.8)	867 (4.8)	829 (0.2)	662 (−20.0)	569 (−31.2)	759 (−8.2)	826 (−0.1)	845 (2.2)	752 (−9.1)	839 (1.5)

ICD-10: International statistical classification of diseases and related health problems, 10th revision; M:I mortality:incidence.

^a^ Cases/deaths from neighbouring countries were added to compute M:I ratios.

**Table 3:** Cancer incidence in females (Norwegian Cancer Registry, 2010) and estimates using GLOBOCAN methods

Site (ICD-10 code)	No. of observed cases	No. of estimated cases (% difference from observed cases)									
		Method 1A	Method 1B	Method 2	Method 3	Method 4	Method 5	Method 6	Method 7	Method 8	Method 9
Head and neck (C00–14)	188	174 (−7.5)	187 (−0.5)	163 (−3.3)	165 (−12.2)	136 (−27.7)	157 (−16.5)	155 (−17.6)	174 (−7.5)	192 (2.1)	185 (−1.6)
Oesophagus (C15)	58	62 (6.9)	56 (−3.5)	56 (−3.5)	56 (−3.5)	56 (−3.5)	61 (5.2)	53 (−8.6)	64 (10.3)	54 (−6.9)	85 (46.6)
Stomach (C16)	196	201 (2.6)	218 (11.2)	230 (17.4)	210 (7.1)	188 (−4.1)	209 (6.6)	280 (42.9)	200 (2.0)	151 (−23.0)	197 (0.5)
Colon-rectum (C18–21)	1894	1939 (2.4)	1947 (2.8)	1856 (−2.0)	1732 (−8.6)	1647 (−13.0)	2217 (17.1)	1961 (3.5)	1781 (−6.0)	1671 (−11.8)	1309 (−30.9)
Liver (C22)	73	49 (−32.9)	59 (−19.2)	49 (−32.9)	74 (1.4)	65 (−11.0)	86 (17.8)	54 (−26.0)	48 (−34.3)	63 (−13.7)	94 (28.8)
Gallbladder (C23–24)	84	83 (−1.2)	85 (1.2)	80 (−4.8)	44 (−47.6)	23 (−72.6)	34 (−59.5)	72 (−14.3)	79 (−6.0)	75 (−10.7)	96 (14.3)
Pancreas (C25)	358	363 (1.4)	369 (3.1)	355 (−0.8)	361 (0.8)	286 (−20.1)	367 (2.5)	342 (−4.5)	348 (−2.8)	303 (−15.4)	331 (−7.5)
Larynx (C32)	18	10 (−44.4)	14 (−22.2)	15 (−16.7)	17 (−5.6)^a^	18 (0.0)	19 (5.6)	14 (−22.2)	18 (0.0)	19 (5.6)	21 (16.7)
Lung (C33–34)	1210	1197 (−1.1)	1328 (9.8)	1042 (−13.9)	1172 (−3.1)	1026 (−15.2)	1118 (−7.6)	965 (−20.3)	1095 (−9.5)	1173 (−3.1)	1171 (−3.2)
Melanoma of skin (C43)	797	698 (−12.4)	707 (−11.3)	630 (−21.0)	936 (17.4)	1068 (34.0)	1591 (99.6)	557 (−30.1)	675 (−15.3)	793 (−0.5)	646 (−19.0)
Breast (C50)	2891	3059 (5.8)	2896 (0.2)	2958 (2.3)	2672 (−7.6)	2550 (−11.8)	4540 (57.0)	2818 (−2.5)	3062 (5.9)	2772 (−4.1)	3651 (26.3)
Cervix (C53)	307	285 (−7.2)	296 (−3.6)	310 (1.0)	282 (−8.1)	234 (−23.8)	338 (10.1)	330 (7.5)	312 (1.6)	286 (−6.8)	250 (−18.6)
Corpus uteri (C54)	744	801 (7.7)	763 (2.6)	715 (−3.9)	639 (−14.1)	632 (−15.1)	691 (−7.1)	721 (−3.1)	740 (−0.5)	721 (−3.1)	599 (−19.5)
Ovary (C56)	479	432 (−9.8)	462 (−3.6)	489 (2.1)	451 (−5.8)	445 (−7.1)	559 (16.7)	477 (−0.4)	509 (6.3)	457 (−4.6)	392 (−18.2)
Kidney (C64–66)	246	269 (9.3)	272 (10.6)	237 (−3.7)	215 (−12.6)	140 (−43.1)	263 (6.9)	232 (−5.7)	229 (−6.9)	216 (−12.2)	243 (−1.2)
Bladder (C67)	359	378 (5.3)	326 (−9.2)	349 (−2.8)	351 (−2.2)	321 (−10.6)	438 (22.0)	353 (−1.7)	347 (−3.3)	329 (−8.4)	306 (−14.8)
Brain (C70–72)	629	903 (43.6)	858 (36.4)	688 (9.4)	663 (5.4)	407 (−35.3)	788 (25.3)	672 (6.8)	672 (6.8)	542 (−13.8)	459 (−27.0)
Thyroid (C73)	206	184 (−10.7)	176 (−14.6)	163 (−20.9)	189 (−8.3)^a^	181 (−12.1)	236 (14.6)	177 (−14.1)	152 (−26.2)	206 (0.0)	242 (17.5)
Hodgkin lymphoma (C81)	57	56 (−1.8)	54 (−5.3)	51 (−10.5)	25 (−56.1)^a^	23 (−59.7)	39 (−31.6)	56 (−1.8)	50 (−12.3)	54 (−5.3)	54 (−5.3)
Non-Hodgkin lymphoma (C82–85, C96)	412	398 (−3.4)	390 (−5.3)	374 (−9.2)	292 (−29.1)	274 (−33.5)	436 (5.8)	371 (−10.0)	369 (−10.4)	381 (−7.5)	385 (−6.6)
Multiple myeloma (C88, C90)	159	164 (3.1)	164 (3.1)	162 (1.9)	126 (−20.8)	130 (−18.2)	207 (30.2)	151 (−5.0)	171 (7.6)	151 (−5.0)	129 (−18.9)
Leukaemia (C91–95)	272	260 (−4.4)	253 (−7.0)	248 (−8.8)	216 (−20.6)	197 (−27.6)	307 (12.9)	223 (−18.0)	265 (−2.6)	276 (1.5)	218 (−19.9)
Other and unspecified	829	910 (9.8)	857 (3.4)	855 (3.1)	762 (−8.1)	726 (−12.4)	921 (11.1)	807 (−2.7)	886 (6.9)	767 (−7.5)	876 (5.7)

ICD-10: International statistical classification of diseases and related health problems, 10th revision; M:I mortality:incidence.

^a^ Cases/deaths from neighbouring countries were added to compute M:I ratios.

Among the methods commonly used in low- and middle-income countries (methods 5 to 9), the method using mortality combined with 5-year relative survival proportion (method 5) produced quite large overestimates for cancers associated with good survival including melanoma of skin in women (+99.6%; +794 cases), prostate (+92.8%; +4208 cases) and breast (+57.0%; +1649 cases) and underestimates for cancers with small numbers of deaths, including testicular (−40.3%; −116 cases) or gallbladder (−61.6%; −45 cases in men, −59.5%; −50 cases in women) cancers. Estimates for lung and pancreatic cancers were similar to, or more accurate than, those obtained from method 3 and 4 (Table 2 and Table 3).

The performance of methods using data from one or more regional registries (methods 6 and 7) varied greatly by cancer site. Estimates for prostate, colorectal, lung and breast cancers were reasonable (less than 8% difference between estimates and observed data); method 6, however, underestimated female lung cancer estimates in our study (−20.3%; −245 cases). Despite the use of observed data (instead of GLOBOCAN estimates), results from methods 8 and 9 were also almost exclusively underestimates and their accuracy varied greatly by cancer site and sex (Table 2 and Table 3).

## Discussion

Our results, validated against the high-quality data available from the Norwegian Cancer Registry, confirm that projections of historical national data are among the best methods to predict recent cancer incidence. They also suggest that, in selected populations, a site-specific approach is warranted for cancers where the level of incidence is driven by changes in diagnosis patterns (e.g. thyroid) or screening (e.g. breast, prostate). They also illustrate how the accuracy of national estimates based on geographic proxies – including data from regional registries or neighbouring countries – is highly dependent on the extent to which these datasets are representative of the scale and profile of the country of interest.

In Norway, where long-term national cancer incidence data series are available, the projection of historical rates^9^ (method 1A) resulted in a relatively good estimation of recent incidence statistics. Projections-based methods captured medium- to long-term trends reasonably but did not perform as well when there were recent changes in the trends. For example, prostate cancer rates increased by 4.3% annually in Norway between 1985 and 2008^13^ but plateaued in recent years.^14^ Thus method 2, which simply applies the most recent cancer incidence rates available to recent population data, performed better than a projection of historical rates in this context (Fig. 5). On the other hand, lung cancer rates have uniformly increased in Norwegian women^15^ in recent years, explaining the good quality of estimates based on trends for this cancer (Fig. 6).

Applied to the Norwegian data, methods 3 and 4 were less accurate than the first two methods and underestimated the overall number of cases. They were notably less reliable for cancer sites with small numbers of deaths such as thyroid (males) or testicular cancers. Although the incidence of testicular cancer has uniformly increased in Norway over recent decades, mortality from this cancer has declined since the late-1970s, leading to low numbers of annual deaths (13 deaths nationally in 2010).^14^ In this context, methods 3 and 4 failed to accurately estimate incident cases in age groups where deaths are rare and tend to underestimate the overall cancer burden. Furthermore, these methods also depend on the representativeness of the proxy datasets used to compute the M:I ratios on which they rely.

In GLOBOCAN 2012, method 5 was mainly used in the Caribbean, Latin America and some Asian countries. Applied to Norway, it performed equivalently or better than methods 3 and 4 in cancers with a poor prognosis such as lung or pancreatic cancer, for which the 5-year relative survival proportion in Norway is 15% and 6%, respectively, for male diagnoses 2009–2012.^14^ However, the method was inadequate for cancers with good prognosis such as melanoma, breast or prostate cancers, where the 5-year relative survival rate was above 80%.^14^ For the latter two cancers, cure is not apparent at 5 years and survival proportions continue to decline in further years of follow-up,^16^ thus invalidating the equation used to calculate incidence (Equation 1).

It is likely that method 5 combined with longer-term relative survival estimates would produce better incidence estimates for cancers with a good prognosis. In Norway, 10-year relative survival proportions for prostate and breast cancers are available and reduced to 58% and 71%, respectively.^17^^,^^18^ However, such data are less frequently available than 5-year relative survival proportions, particularly in countries were method 5 would be applied. In many low- and middle-income countries, where curative treatments may not be available and hence the M:I ratio is higher, the 5-year survival proportion may be a better proxy of case-fatality. For example, 5-year relative survival proportions for breast cancer in Costa Rica was 68% for diagnoses 1995–2000^19^ while the M:I ratio was 31.8% based on data from 1998–2002,^20^ indicating that method 5 would produce reliable estimates in this setting.

Because of the paucity of cancer data, national incidence in low- and middle-income countries is often estimated using datasets from regional registries or neighbouring countries. Most of the GLOBOCAN 2012 estimates for Africa and south-east Asia were based on such data (methods 6 to 9). Applying these methods to the Norwegian data illustrated the problem of a lack of representativeness of proxy datasets used to derive national cancer incidence. For example, method 7, where data from the country’s capital city were used, provided relatively good overall estimates for Norway. In many low-income countries, the differences are likely to be considerably greater where there are marked differences in the profile of cancer in rural and urban settings. As an example, the breast cancer rate in Mumbai, India (a major urban area) was 31.0 per 100 000 person-years in 2008–2009, more than 2.5 times the rate observed in Barshi (12.3 per 100 000), a rural area, in 2009–2010.^21^

Producing accurate national cancer incidence estimates is a difficult task that depends on multiple factors: the availability of high-quality cancer registry data, the use of valid and reproducible estimation methods and the representativeness of proxy datasets used for calculations. Because this study was performed using high-quality cancer registry data from a high-income country, the impact of data quality issues and regional variations of the cancer burden on our results are likely to be minimal. The findings should mainly reflect the intrinsic characteristics of the different methods of estimation. On the other hand, it also means that our site- and method-specific results cannot be generalized to other countries and may not be valid in different settings. However, our study provides general conclusions regarding the context in which the different methods are likely to produce reliable estimates, provided that the required data are available.

The study provides a comparative assessment of the different methods of estimation of national incidence used in GLOBOCAN as well as some general guidance on the caveats associated with certain methods of estimation for specific cancer types. In particular, they indicate that in countries such as Norway with longstanding high quality population-based cancer registries, regional-based or trends-based estimates perform reasonably well in comparison with recorded incidence. However, such an evaluation of the validity of the estimates themselves is only possible in a few countries with high-quality national data. Elsewhere, data quality issues or a lack of national representativeness of regional datasets could potentially undermine the validity of the estimates and the evidence-based evaluation process. Assessment of uncertainty would also require additional adjustment for the completeness, accuracy and representativeness of the source information.

Along with the continuous assessment and improvements of estimation methods, efforts should be targeted at supporting the development of cancer registration worldwide. The Global Initiative for Cancer Registry Development^22^ is a global partnership launched in 2011 with a goal to increase the coverage and quality of registries in low- and middle-income countries. The partnership plays a critical role in capacity-building, to attain more robust data for national and global cancer estimation purposes and aid countries in the prioritization and evaluation of national cancer control plans.
